# Clinical diagnosis and treatment recommendations for ocular toxicities of targeted therapy and immune checkpoint inhibitor therapy

**DOI:** 10.1111/1759-7714.13327

**Published:** 2020-02-04

**Authors:** Xiaowei Liu, Zheng Wang, Chan Zhao, Hanping Wang, Xiaoxiao Guo, Jiaxin Zhou, Lian Duan, Xiaoyan Si, Li Zhang, Yue Li, Mengzhao Wang, Meifen Zhang, Li Zhang

**Affiliations:** ^1^ Department of Ophthalmology Peking Union Medical College Hospital, Peking Union Medical College and Chinese Academy of Medical Sciences Beijing China; ^2^ Department of Ophthalmology Beijing Hospital Beijing China; ^3^ Department of Pulmonary and Critical Care Medicine Peking Union Medical College Hospital Beijing China; ^4^ Department of Cardiology Peking Union Medical College Hospital Beijing China; ^5^ Department of Rheumatology Peking Union Medical College Hospital Beijing China; ^6^ Department of Endocrinology Peking Union Medical College Hospital Beijing China; ^7^ Department of Clinical Laboratory Peking Union Medical College Hospital Beijing China; ^8^ Department of Gastroenterology Peking Union Medical College Hospital Beijing China

**Keywords:** Immune checkpoint inhibitor, immune‐related adverse events (irAEs), ocular toxicities, targeted therapy

## Abstract

The increased use of targeted therapy and immune checkpoint inhibitors in cancers has brought new hope of survival to patients with advanced tumors. However, increasing numbers of immune‐related adverse events (irAEs) of these medications have been reported, affecting almost all human organs including the eye. These adverse effects may affect the entire ocular region, including the eyelid, eye lashes, conjunctiva, cornea, uvea, retina and optic nerve, and have thus far been largely ignored by patients and doctors. In this review, we summarize the characteristics of ocular diseases related to irAEs and advise on how to diagnose and manage these diseases.

**Key points:**

This review will enable clinical oncologists to recognize, diagnose, and manage targeted therapy and immune checkpoint inhibitor‐related ocular adverse events.

## Introduction

Targeted therapy and immune checkpoint inhibitors are sometimes the last hope for advanced cancer patients. The former kills the tumor directly and the latter kills the tumor by enhancing T cells via cytotoxic T lymphocyte associated protein (CTLA‐4) and programmed cell death protein‐1 (PD‐1) and its ligand. As these therapies are increasingly administered to patients clinically, various forms of immune‐related adverse events (irAEs) including ocular toxicities have been reported. Ocular toxicities are uncommon but may cause severe threats to sight and reduce a patient's quality of life. Furthermore, such side effects may affect patient compliance with treatment. While neither ophthalmologists nor oncologists know much about ocular irAEs, patients pay even less attention to these conditions.

Ocular toxicities such as blurring of vision and ocular discomfort have been reported in phase I or phase II clinical studies of immune checkpoint inhibitors.^1^ Since then, increasing reports of ocular toxicities have been published, including blepharitis, conjunctivitis, uveitis,^2^, ^3^, ^4^ scleritis^5^ and choroidal retinitis,^6^ while the pathological mechanisms remain unelucidated.

Among the reported medications, anti‐CTLA4 (ipilimumab) had an ocular toxicity rate of 1.3%,^7^ including anterior uveitis, optical neuropathy, Grave's syndrome‐like oculopathy and Vogt‐Koyanagi‐Harada (VKH) like syndrome.^8^ Vemurafenib had an ocular side‐effect rate of 4%, which mostly comprised uveitis,^8^ while anti‐PD‐1 was reported to have side‐effects of blurred vision and tearing.^1^, ^9^

In this article, we report and review the ocular toxicities caused by targeted therapy and immune checkpoint inhibitors and discuss the underling pathogenesis, diagnosis and treatment policies.

## Toxicities of the eyelids, eyebrows and eyelashes

These toxicities most commonly occur in EGFR inhibitor‐treated patients. Almost all ocular tissues share the same EGFR that drives cancer growth, including the meibomian gland, follicles, conjunctiva, cornea, lacrimal gland, eyelid skin and the microvascular system. Therefore, targeting of this receptor is likely to cause various toxicities.

### Dermatitis of the eyelid

Patients treated with anti‐EGFR frequently complain of dermatitis, including that of the face and eyelids. This dermatitis demonstrates the same clinical features as other skin rashes caused by anti‐EGFR antibody, including tiny rashes scattered over the face and eyelids, most of which are symptomless, while a few cause itching and discomfort. No treatment is usually needed.

### Trichomegaly of the eyebrows and eyelashes

Overgrowth of the eyelashes and eyebrows is a common finding in anti‐EGFR‐treated patients. While lengthened eyelashes may be appealing to some, the affected hair is always curled and unruly, thus with potential to irritate the cornea and cause discomfort. Furthermore, additional facial hair may be particularly distressing for female patients. However, no treatment is needed for most patients.

### Entropion or ectropion

Entropion and ectropion has also previously been reported^10^ although the underlying pathology is unknown. However, it may be just coincidental with involutional ectropion or ectropion. Surgery is the only way to treat these conditions.

## Blepharitis and conjunctivitis

Blepharitis and conjunctivitis have been diagnosed among both targeted and immune checkpoint inhibitor therapy patients.^10^

### Symptoms

Itching, chronic eyelid redness, eye irritation, dry, burning sensation, photophobia and increased lacrimation and mucoid discharge are the most common symptoms caused by blepharitis and conjunctivitis.

### Signs

Pachyblepharon, red eyelid margin, conjunctival hyperemia and scurf or crusting around the eyelashes might be seen in patients with blepharitis. The dilated and congested opening of the meibomian gland would be seen, sometimes with keratinization on visualization under slit‐lamp microscopy.

### Diagnosis

Blepharitis can be diagnosed by the symptoms and typical signs including eyelid margin redness, scurf or/and crusting around the lashes.

### Management

Eyelid hygiene and application of a warm compress may help reduce bacterial colonization and the accumulation of sebaceous secretions, and are commonly used to manage this condition. Anti‐inflammatory ointment may also be applied. Associated dry eye is very common^11^ in patients with blepharitis, so artificial tears are always necessary.

### Prognosis

The symptoms may be relieved quickly with proper margin cleaning and medications, but recurrence is common when cleaning of the margin and medication ceases.

## Dry eye

In clinical trial reports of CTLA4 and PD‐1‐targeting antibodies, there was incidence of dry eye of 1.2%–24.2%,^11^ the underlying pathology of which has not yet been fully elucidated. Furthermore, the condition is often ignored due to the high incidence of dry eye in the normal population.

### Symptoms

Dryness, pain, foreign body and burning sensations, photophobia, blurred vision, red eye and eye strain are the most common complaints in dry eye patients.

### Signs

Signs of dry eye include conjunctiva hyperemia; decreased tear meniscus and debris in the tear film; epithelial keratopathy, which can be demonstrated following the instillation of fluorescein, rose Bengal or lissamine green; rapid tear break‐up time (TBUT) of less than 10 seconds, normally more than 10 seconds among healthy individuals; reduced tear secretion—in dry eye patients the value is usually less than 10 mm/5 minutes, while the normal value is 10–15 mm/5 minutes.

### Diagnosis

The diagnosis of dry eye should combine evidence gleaned from symptoms, tear stability as determined by TBUT, and ocular surface damage, ascertained by staining with the dyes mentioned above.

### Management

While uncomfortable, this condition is readily managed by various means including life style changes, to avoid prolonged periods of mobile phone or computer screen reading; application of lubrications, of which artificial tears are the most frequently used medication for dry eye patients with use of preservative‐free tear substitutes being recommended; administration of anti‐inflammatory therapy, topical low‐dose corticosteroids and cyclosporine A. In patients with severe dry eye or for those eyedrops were not available, punctual occlusion may be beneficial for sustaining tears for longer on the ocular surface. Good eyelid margin hygiene, warm compress and lid massage are also needed in patients with meibomian gland dysfunction to liquify the thickened secretions and facilitate outflow.

### Prognosis

Topical eyedrops, eyelid hygiene and massage are responded to and tolerated well by patients. However, long‐term therapy is needed as recurrence inevitably occurs when medication is stopped.

## Keratitis

The pathogenesis of this condition might be associated with destruction of the corneal epithelial cells' healing potential, causing prolonged corneal epithelium defects.^10^

In 2009, Johnson *et al* 12 reported a case of persistent corneal erosion that finally deteriorated into infective keratitis during erlotinib therapy. The keratitis was not resolved until two weeks after discontinuation of erlotinib. Several other reports have also described failure of all treatment efforts until withdrawal of the immune therapy.^13^, ^14^

### Symptoms

Keratitis is characterized by pain, photophobia, tearing, eye redness and impaired vision.

### Signs

Conjunctival and limbus injection, corneal epithelial erosion, and corneal ulcer were the most common signs reported. In some severe cases, hypopyon and even corneal perforation and atrophy of the eyeball might occur.

### Diagnosis

Diagnosis can be made based on the clinical features and findings. However, secondary infection by microorganisms may make diagnosis of keratitis as an irAE more difficult.

### Management

Antibiotic eye drops to prevent secondary infections can be used. Lubrication or autologous serum eye drops can be administered to promote corneal healing, while cycloplegia may be needed in severe patients. Use of a bandage contact lens is helpful for corneal would healing, but when infection is suspected, contact lens use is not recommended. Finally, discontinuation of the anticancer therapy must be considered by both doctors and patients if the corneal ulcer remains unresolved despite all efforts made.

### Prognosis

Mild keratitis may resolve quickly after proper treatments without vision impairment, but severe corneal ulcer may result in vision loss and even loss of the eyeball.

## Scleritis (episcleritis and scleritis)

The incidence of scleritis as an irAE is less than 1%,^15^ including both episcleritis and scleritis. The former is superficial and localized, while the latter is deeper and diffuse.

### Symptoms

Scleritis is typically a sudden onset, self‐limited disease with pain, eye redness and irritation. The pain varies from very mild to nontouchable according to the severity of the disease.

### Signs

Eye redness in scleritis can be pink, bright red for episcleritis or of violaceous hue for scleritis; it can also be localized or diffuse with dilated and injected blood vessels. Nodules might be seen localized in the exposed interpalpebral zone of the eye, known as nodular scleritis. When the posterior sclera is involved, papilledema and thickening of the choroid and exudative retinal detachment might be found. Necrotizing scleritis is the most severe type but has not yet been reported in irAE studies.

### Diagnosis

Diagnosis is made based on clinical evidence including localized or diffuse eye redness, pain and tenderness to touch. B‐scan ultrasound, computed tomography and magnetic resonance imaging (MRI) may also be helpful in establishing the diagnosis.

### Management

Episcleritis may clear without treatment but topical nonsteroidal anti‐inflammatory drugs (NSAIDs) may help relieve the pain. In cases that do not respond to NSAIDs, topical steroids may be necessary and beneficial. Oral NSAIDs may also be helpful and effective to relieve the pain caused by scleritis. Oral or intravenous corticosteroids may be needed in posterior scleritis and some severe and persistent cases. For refractory patients who respond poorly to corticosteroids, systemic immunosuppressive therapy (e.g., methotrexate, cyclosporine or cyclophosphamide) is recommended.

### Prognosis

Most patients respond well to topical corticosteroids. Importantly, for severe scleritis such as necrotizing scleritis, delayed therapy might result in blindness and loss of the eyeball.

## Uveitis

The uveal tract comprises the iris, ciliary body and choroid. Inflammation of any component is known as uveitis. The uveal tract is a highly vascular, heavily pigmented tissue, and is prone to autoimmune disorders. CTLA4 and PD‐1 antibodies upregulate the functions of T cells, which may cause T cell‐associated autoimmune diseases like uveitis.^16^ Also, in a clinical trial of tumor infiltrating lymphocytes plus interleukin 2 (TIL + IL2), 35 patients were enrolled and five were diagnosed with uveitis after treatment. They were treated successfully by topical corticosteroids.^17^, ^18^ In most reported cases, uveitis as an irAE developed within 12 weeks of receiving immune therapy, and was relieved in 6–8 weeks after proper treatment.^8^

Uveitis can be classified into anterior‐, intermediate‐, posterior‐ and pan‐uveitis. IrAEs may cause all types of uveitis mentioned above.^16^

### Symptoms

Reported symptoms include ocular pain, photophobia and tearing, blurred vision, floaters and headache.

### Signs

Signs include perilimbal conjunctival injection; miosis (small pupil); keratic precipitates (KP), an inflammatory cellar deposit on the inner surface of corneal endothelium, which can be large, white and greasy, thus known as mutton‐fat KP, or if very small, called dust KP; anterior chamber flare and cells due to protein and inflammatory cells that have infiltrated or leaked into the anterior chamber from the inflamed vessels—in severely‐affected patients, fibrinous exudate or hypopyon might be formed; posterior synechiae, which causes the pupil to become an irregular shape; increased intraocular pressure due to the inflammation of the trabecular meshwork; pigment deposits on the anterior surface of the lens; cells and flare in the vitreous; choroidal retinitis, including the signs of optic disc edema, retinal edema, cotton‐wool spots and hemorrhage in the retina, macular edema and even exudative retinal detachment; vasculitis of the retina and/or choroid.^8^, ^19^

### Work‐up

Ultrasonography is a key tool in diagnosis of uveitis. Patients with uveitis often have media opacification that limits direct visualization of the fundus. Ultrasonography can provide important information regarding the vitreous, retina and optic nerve.

Fluorescein angiography (FA) and indocyanine green angiography (ICGA) are essential methods for posterior uveitis diagnosis. These techniques can provide detailed images of vascular changes in the retina and choroid, while optical coherence tomography (OCT) is highly sensitive to macular edema and subretinal fluid caused by posterior uveitis.

### Diagnosis

The diagnosis of uveitis is made based on the history of targeted or immune checkpoint inhibitor therapy and clinical findings combined with the results of essential examinations such as ultrasonography, MRI, ICGA, FA and OCT.

Clinical findings including symptoms of red eye, pain, blurred vision, photophobia and signs of perilimbal injection, KP, cells and flare in the anterior chamber, posterior synechiae may help to make a diagnosis of anterior uveitis.

Cells and floaters in the vitreous, papillary edema, retinal edema, retinal vasculitis and macular edema are always suggestive of posterior uveitis. Intermediate uveitis usually has a quiet and insidious onset, with floaters or mild photophobia as symptoms, minimal anterior chamber reaction and white or yellow‐gray exudates at the ora serrata area.

### Management

Mydriasis and cycloplegia can prevent the formation of iris adhesions posteriorly to the lens or anteriorly to the cornea and can also relieve the photophobia from iris sphincter spasm and painful ciliary muscle action associated with iridocyclitis. Topical or systemic corticosteroid therapy is the mainstay of therapy for uveitis and most patients response well to this.^8^, ^20^ For patients with anterior and mild intermediate uveitis, topical preparations are usually effective, while in patients with posterior uveitis caused by irAEs, systemic corticosteroids are needed, usually starting from 1 mg/kg and tapering according to the clinical response. In addition to topical or systemic corticosteroids, triamcinolone periocular space injection is also an effective therapy choice.^16^

Cytotoxic and immunosuppressive agents may also be used in patients who respond poorly to appropriate corticosteroid therapy or those with severe sight‐threatening inflammation. This should always be combined with prednisolone. Additionally, NSAIDs may also be administered, while other treatment choices such as retinal photocoagulation or vitrectomy surgery are available to treat complications.

### Prognosis

The prognosis is generally good for those who receive prompt diagnosis and treatment, but complications including cataracts, glaucoma, band keratopathy, macular edema and permanent vision loss may result if left untreated. Therefore, for refractory cases, the benefit of discontinuing the immune therapy should be evaluated by oncologists, ophthalmologists together with the patient.

## Vogt‐Koyanagi‐Harada like syndrome

VKH like syndrome is a special kind of uveitis and is the most commonly reported irAE in immune checkpoint inhibitor therapy. As such, it merits discussion as a distinct entity.

VKH is a rare multisystem condition implicating a systemic autoimmune reaction against melanocytes. Organs with melanocytes might be involved, including the uveal tract, inner ear, skin and the meninges. Bilateral pan‐uveitis with exudative multifocal retinal detachments associated with vitiligo, poliosis, alopecia, tinnitus, hearing loss, meningism with headache and pleocytosis of cerebrospinal fluid are the classic clinical findings.

The autoimmune reaction against melanocytes in VKH is mainly mediated by T lymphocytes, and the antitumor effects of immune checkpoint inhibitors are also achieved by enhancing T cell function, suggesting that they might be the same T cell clone.^21^ Interestingly, CTLA4 and PD‐1 genetic polymorphisms have been found among VKH patients.^22^ CTLA4 and PD‐1 antibodies may disrupt the balance of tumor cell killing and immune tolerance to melanocytes via CTLA4 or PD‐1 signals and therefore be involved in the pathogenesis of VKH.

It has been reported that CTLA4^23^ or PD‐1 antibodies,^24^, ^25^ or a combination of the two^26^, ^27^ might cause VKH‐like syndrome, as does TIL + IL2 therapy.^17^ Patients suffering from VKH‐like syndrome were initially diagnosed with mostly metastatic melanoma^25^ and a few with non‐small cell lung cancer.^24^

### Symptoms and signs

Typically in VKH, four stages with different clinical features occur.

**Figure 1:**
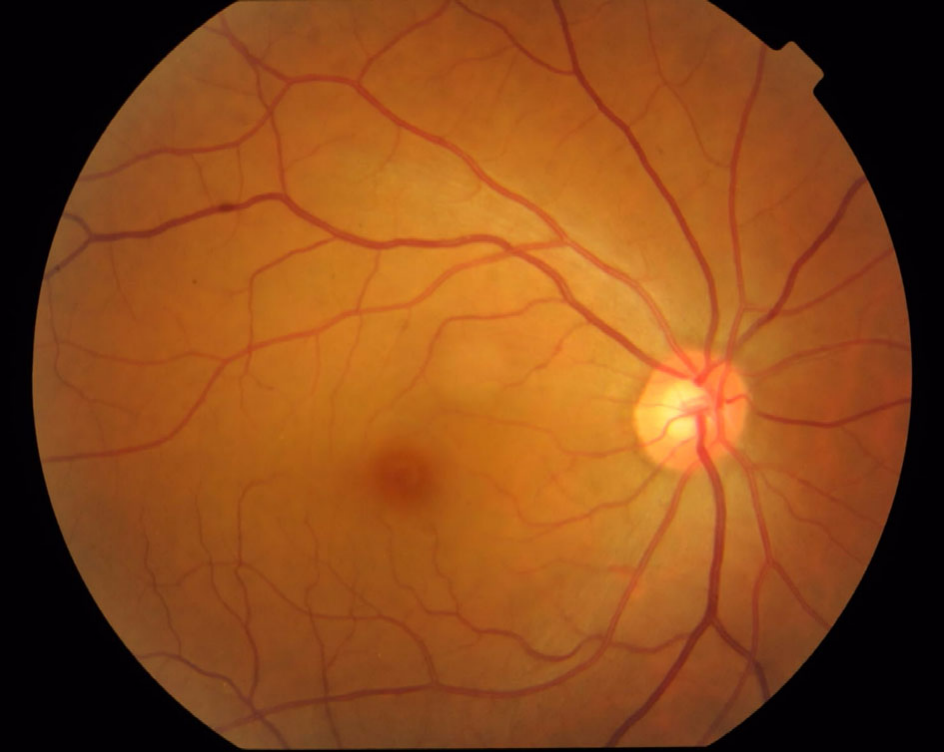
Normal fundus.

**Figure 2:**
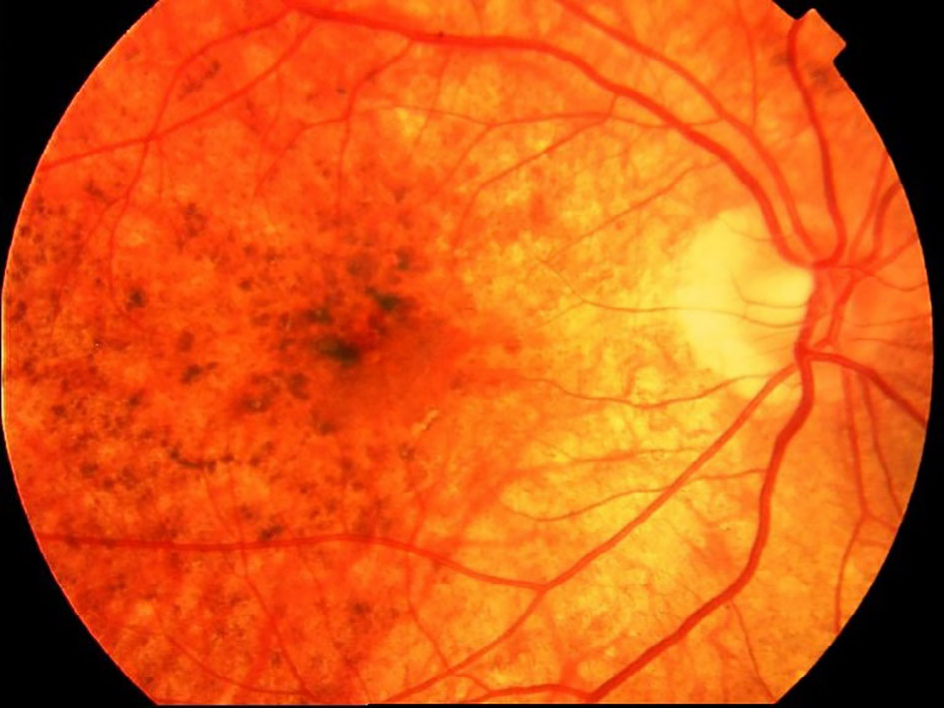
“Sunset glow fundus” in a VKH patient.

**Figure 3:**
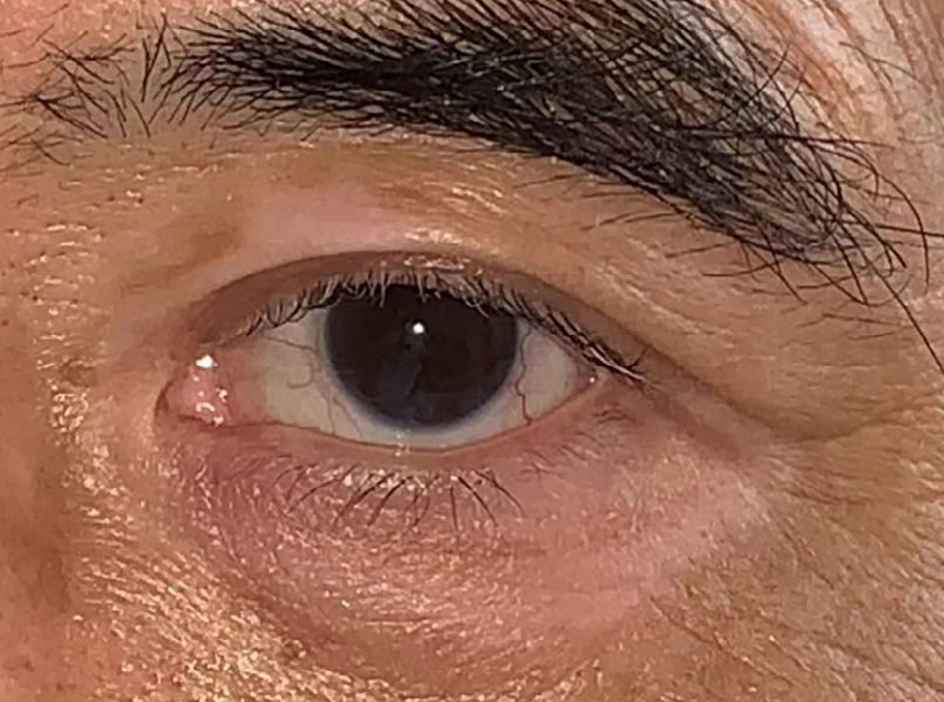
Eyelid skin vitiligo and poliosis of the eyelashes in a VKH patient.

1. **Prodromal stage** In this stage the patient might have a nonspecific viral‐like illness with fever, nausea, dizziness, headache, tinnitus, retrobulbar pain and meningism for a few days.
2. **Acute uveitic stage** Bilateral blurry vision is the main complaint in this stage, with signs of posterior uveitis and multiple serious retinal detachments, optic nerve head hyperemia and edema.
3. **Convalescent stage** The hallmark of this stage is choroidal depigmentation with classic sunset glow fundus (Fig 1, 2) with Dalen‐fuchs nodules. The patient may also have vitiligo and poliosis a few months after the acute stage (Fig 3).
4. **Chronic recurrent stage** Recurrent anterior uveitis is characteristic of this stage, while posterior uveitis, choroidal neovascular membranes, secondary cataract and glaucoma might also occur.

Compared with typical VKH, symptoms and signs vary greatly in VKH‐like syndrome associated with irAEs. They can be almost the same as typical VKH,^27^ but untypical clinical features are more common.^17^ For example, incomplete symptoms and signs might occur in VKH‐like syndrome, such as patients having no symptoms of meningism, hearing loss or vitiligo.^19^ Furthermore, differences in the sequence of symptoms and signs may occur. Patients might have no prodromal stage and the vitiligo might occur before the ocular signs.^17^, ^25^ Similarly, differences in the severity of symptoms may be experienced, as in some cases it has been reported there were no anterior signs but only serum exudative retinal detachments,^26^ while severe anterior inflammation but mild retinal reaction has been recorded in other patients. Other cases have already demonstrated sunset glow fundus when referred without any prodromal signs or symptoms.^21^ Finally, the time of VKH‐like syndrome onset after immune therapy varied greatly, ranging from two weeks to 13 months.

### Diagnosis

**Figure 4:**
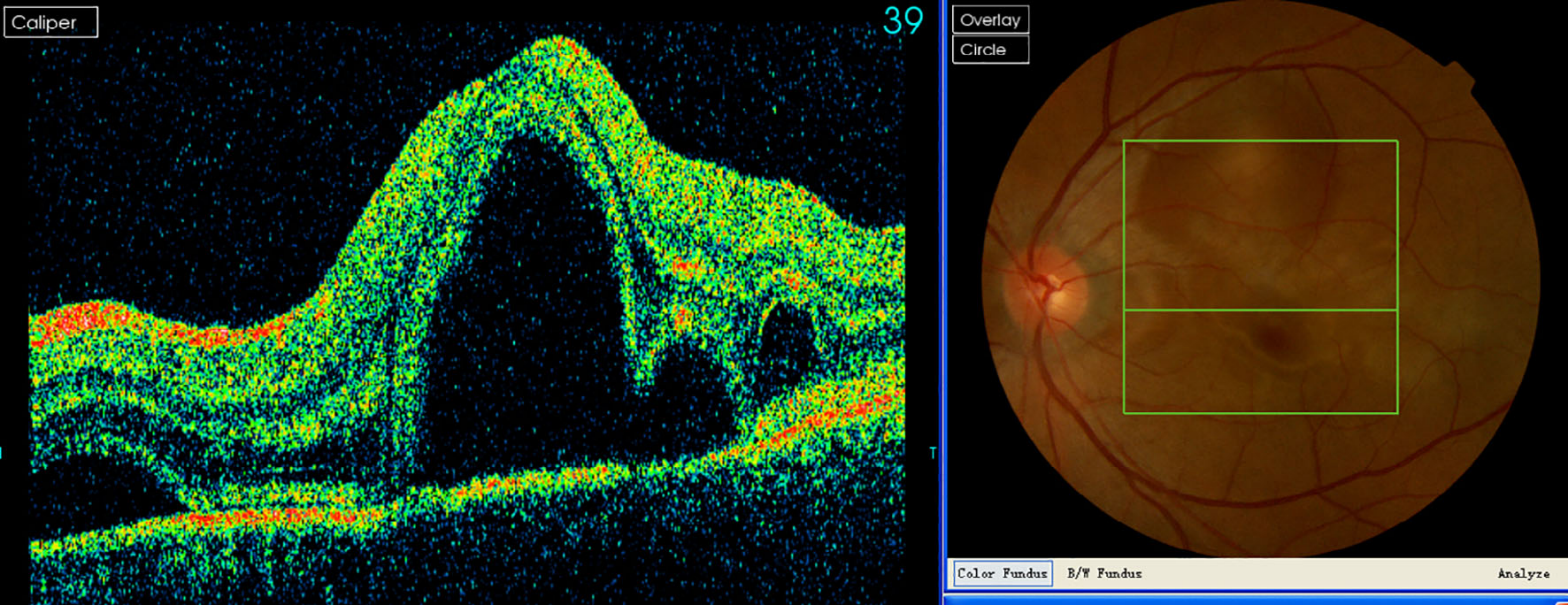
Retinal serum detachment of the central retina in a PD1 antibody‐treated patient.

Diagnosis is based on clinical findings, but OCT, FA and ICGA may be helpful in detecting subretinal fluid and serum retinal detachments (Fig 4). However, it must be remembered that multiple serum retinal detachments could also be found in metabolic carcinoma, especially lung cancer. The choroid is one of the most common sites for metabolic tumors, which might raise difficulties for differentiation. Pleocytosis might be found in a cerebrospinal fluid test.

### Management

Management of this condition is the same as previously described for uveitis, including mydriasis and cycloplegia, corticosteroids, NSAIDs, immunosuppressive agents, retinal photocoagulation and vitrectomy surgery.

### Prognosis

Prognosis of VKH‐like syndrome as an irAE depends on the response to topical or systemic corticosteroids. In patients with severe and sight threatening inflammation and poor response to proper therapy, discontinuation of the causative immune therapy should be considered.

## Graves‐like eye disease

Graves eye disease (GED) is also called thyroid‐associated orbitopathy and Graves ophthalmopathy. It is an inflammatory orbit disease associated with autoimmune thyroid disorders. Pathogenesis is related to abnormal expression of thyrotropin receptor antibody (TRAB) in ocular tissue and T cell immunology. As such, Graves‐like eye disease may occur and indeed has been reported following immunotherapy with CTLA 4 and PD‐1 antibodies.^26^

### Symptoms

Symptoms include bilateral proptosis and eyelid swelling; enlarged and swollen extraocular muscles, fat and other soft tissues causing protrusion of the eyeball, which can be bilateral or lateral and asymmetric; eye pain, especially upon movement of the eyes; redness; diplopia and restricted eye movements caused by extraocular swelling and inflammation in the early stages, while fibrosis occurs in the later stage; blurred vision, usually caused by compressive optic neuropathy.

### Signs

Several signs indicative of Graves‐like eye disease can be observed. Among these, eyelid retraction and lid lag in downgaze are the most common clinical features. Proptosis and restrictive strabismus may also be observed, as are conjunctiva edema and corneal erosion. Chemosis, conjunctival injection and caruncular edema may be caused by inflammation and lagophthalmos. Enlargement of the extraocular muscles and fat results in crowding of the orbital apex, which may cause compressive optic neuropathy.

### Diagnosis

Diagnosis is made based on the history of immune therapy; blood tests for thyroxine, thyroid‐stimulating hormone and TRAB levels; classic clinical findings such as eyelid retraction and proptosis; radiographic findings of enlarged rectus muscles.

### Management

Other than restoring thyroid function, local lubrications might work in mildly affected patients without any other treatment. In moderate to severe active disease, glucocorticoids are recommended. In sight‐threatening compressive optic neuropathy that responds poorly to medication, decompressive surgery is recommended. Discontinuation of the causative immunotherapy may also be considered when sight is threatened.

### Prognosis

GED usually has a relatively good outcome after proper systemic glucocorticoid therapy.

## Periorbital edema and cystoid macular edema

Imatinib and other agents such as dasatinib and nilotinib have been reported by Renouf *et al*. to cause periorbital fluid accumulation in approximately 30% of patients and also cystoid macular edema.^10^ The treatment recommendation in this report was also general corticosteroids.

## Exudative retinal detachment

Anti‐VEGF antibodies are currently approved for clinical use in a variety of cancers. While intravitreal injection is now widely used for the treatment of several ocular disorders including diabetic retinopathy, age‐related macular degeneration, retinopathy of prematurity, and retinal vein occlusion complications, the systemic use of these agents seems to be associated with risk of direct ocular toxicity such as exudative retinal detachment.^28^ In addition, these agents increase the risk of systemic hypertension, which could also potentially affect the vascular supply to the visual pathway and cortex.^10^ The underling pathogenesis, while not yet fully elucidated, does not appear to be through the VEGF signal but may be via other factors in this pathway.^28^

## Choroidal neovascularization

In 2013, Modjtahedi *et al*. reported an 81‐year‐old male patient with metabolic melanoma who accepted ipilimumab therapy.^29^ A year later, he complained of vision loss, and macular edema and choroidal neovascularization were confirmed by FA and OCT. He accepted anti‐VEGF intravitreal injection and his vision recovered after two years' management without stopping ipilimumab.

## Other rare ocular toxicities

Ptosis and paralyzed extraocular rectus muscles caused by autoimmune myositis^30^, ^31^ may occur. Cataract, color distortion, visual disturbance, watering eyes, hemianopsia, and temporal arteritis^9^, ^10^, ^32^ have also been reported.

## Conclusions

In summary, ocular toxicities of targeted and immune therapies have been noted to infrequently occur in many studies and might prove deleterious to a patient's quality of life. Fortunately, most can be cured or relieved by topical or systemic corticosteroids without discontinuing immune therapies. While in severe and refractory patients with severe pain and sight threatening conditions, discontinuing the causative immunotherapy regime has been indicated, overall survival was not affected.^33^ After discontinuation of immune therapy, most of the ocular toxicities could be controlled and cured, but recurrence is likely after restarting targeted or immune checkpoint inhibitor treatment.^28^ However, cessation of immune therapy might cause tumor progression and so must be considered with caution.

A baseline ophthalmologic examination is recommended before starting immunotherapy including visual acuity, intraocular presser, slit lamp examination, retinal photographs, visual field, OCT, electroretinography and visual evoked potential.

Good communication between oncologists and ophthalmologists is essential to protect a patient's ocular and systemic health. The patient should be promptly referred to an ophthalmologist when severe toxicities (severe eye pain, loss of vision) occur.

## Disclosure

The authors confirm that there are no conflicts of interest.
